# Features of Bacterial Microbiota in the Wild Habitat of *Pulsatilla tongkangensis*, the Endangered “Long-Sepal Donggang Pasque-Flower Plant,” Endemic to Karst Topography of Korea

**DOI:** 10.3389/fmicb.2021.656105

**Published:** 2021-07-08

**Authors:** Swarnalee Dutta, Chae Sun Na, Yong Hoon Lee

**Affiliations:** ^1^Division of Biotechnology, Jeonbuk National University, Iksan, South Korea; ^2^Seed Viability Research Team, Baekdudaegan National Arboretum, Bonghwa-gun, South Korea; ^3^Plant Medical Research Center, Advanced Institute of Environment and Bioscience, and Institute of Bio-industry, Jeonbuk National University, Jeonju, South Korea

**Keywords:** endophyte, karst, limestone, microbiome, rhizosphere

## Abstract

Microbes associated with plants significantly influence the development and health of the plants. The diversity and function of microbiomes associated with the long-sepal Donggang pasque-flower (DPF) plant, an endemic and endangered species in karst ecosystems, remain unexplored. In this study, we investigated the features of bacterial communities associated with the rhizosphere and roots of DPF plants and their functions in plant growth promotion. The DPF plants were collected from natural and cultivated habitats, and their 16S rDNA was sequenced to assess the bacterial community structures. The bacterial microbiota was more diverse in wild than in cultivated plants. The core bacterial microbiota commonly functioned as endophytes in both wild and cultivated DPF plants, although there were some differences. The identified bacterial strains benefited plants through nitrogen fixation, phosphate solubilization, or phytohormone production, inducing measurable growth differences in *Arabidopsis thaliana*. To the best of our knowledge, this study is the first to report the bacterial community structures associated with the rhizosphere soil and roots of DPF plants in karst ecosystems. The bacterial strains isolated in this study could be used to aid sustainable growth and restoration of rare plants in karst ecosystems. Our systematic research on the microbiomes associated with these endangered plants will contribute to their conservation as well as development of better cultivation.

## Introduction

Long-sepal Donggang pasque-flower (*Pulsatilla tongkangensis* Y. N. Lee et T. C. Lee; hereafter DPF), a perennial plant species belonging to the family Ranunculaceae, is a rare and endemic species of South Korea at risk of extinction (Korea Biodiversity Information System (KBIS), 2013; Oh et al., 2014). It has a narrow niche width, mostly distributed in the karst topography of Kangwon-do province, South Korea. These plants are primarily found between rocks of limestone located at an altitude of 200–300 m, characterized by alkaline soil (average pH 7.8) with high calcium content. The habitat has relatively low organic material (average 10.51%) and water content (average 19.05%), which together limit the growth of vegetation (Yoo et al., 2009; Oh et al., 2014).

Various factors, including both biotic (plant, microbes, and their interaction) and abiotic (soil type, cultural practices, and ecological environment), influence microbial community structure and diversity in plants and surrounding soils (Micallef et al., 2009; Edwards et al., 2015). The rhizosphere is complex and dynamic, and the unique characteristics of each rhizosphere ecosystem are a result of the complex interactions between microbial communities and plants as well as responses to metabolites produced by the microorganisms and hosts (Waldor et al., 2015). Endophytic microorganisms colonize their hosts internally, and some of them are inherited generationally, while others are acquired from the environment (Hardoim et al., 2008; Dastogeer et al., 2020). Both the rhizosphere and endophytic microbiomes help plants adapt to various adverse environments by alleviating drought, temperature, and salt stresses (Bhattacharyya et al., 2015b; Bhattacharyya and Lee, 2016; Jansson and Hofmockel, 2020). In addition, they suppress plant pathogens by competing for nutrients, producing antibiotics and hydrolytic enzymes, and inducing resistance (Santoyo et al., 2016; Afzal et al., 2019). Therefore, understanding rhizosphere and endophytic microbial ecology is crucial for enhancing plant productivity, stability, and function in ecosystems (Hueso et al., 2011).

Because of habitat destruction and the high value of DPF plants as horticultural resources for garden flowers, governments and private institutes have been attempting to save the species using propagation and transplantation methods (Oh et al., 2014). It has been reported that the rhizosphere and endogenous microbial communities are essential for restoring habitats and ecosystems in the conservation of plants (Zahn and Amend, 2017; Fernández-Gómez et al., 2019). However, we still need more information on how the microbial community structure and diversity are assembled and how they influence the host plant. Comparisons of wild landrace with modern bean accessions showed a significance difference of the rhizosphere bacterial community structures only when grown in agricultural soil, but not native soil (Pérez-Jaramillo et al., 2019). Domestication has led to substantial changes in wheat physiology and disrupted selective processes in the assembly of the wheat microbiome (Hassani et al., 2020). In view of the less diverse microbial communities in cultivated plants compared with their wild relatives, the microbes in wild habitats can be useful sources for the domestication and cultivation of the plants. The microbial community associated with DPF plants can be developed to conserve the plants, but community compositions, functions, and mechanisms of the microbiota have never been explored to date.

In this study, we identified bacterial strains associated with DPF plants growing in the wild in karst ecosystems. The rhizosphere and endophytic bacterial communities were compared with those of cultivated DPF plants. From the analysis, we characterized the core bacterial species associated with the endemic plant species. Furthermore, the bacterial species promoting the growth of model plant *Arabidopsis* were identified, and their features associated with plant growth enhancement were characterized. To the best of our knowledge, this is the first in-depth study to investigate the bacterial community structures associated with DPF plants in the unique limestone environment. The bacterial strains with beneficial functions in this study may be engineered to promote the growth and adaptability of DPF plants in harsh and unique ecological environments.

## Materials and Methods

### Sampling and Sample Processing

Sampling was carried out during the blooming and maturing season (April—May 2020) in an area located at Donggang (Dong River), Jeongsun-gun, Kangwon-do province, northeastern South Korea, which is the specific site for the DPF plants. From each plant collection area, two individual DPF plants were randomly dug out with intact roots along with soil from a 5-cm distance outside of the root zone and combined to form one biological replicate. For each plant, the whole root system was placed in a sterile plastic bag and stored at 4°C. The samples were transported to a laboratory and processed within two days to isolate microbes and DNA from bulk soil, rhizosphere soil, and endosphere (root) samples (Zeng et al., 2018; Fernández-Gómez et al., 2019). For sampling the bulk soil, the root system was gently shaken, and the particles detached during the shaking were collected then sieved through a 2-mm sieve. Subsequently, after removal of the adherent soil, the attached soil was collected as the rhizosphere soil sample using sterilized brushes. The roots were used as endosphere samples after washing with tap water following surface disinfestation with 70% (v/v) ethanol for 1 min, 3% (v/v) sodium hypochlorite solution for 3 min and rinsing five times with sterile water. The surface sterilized roots were blotted with sterile tissue paper to remove excess water. To confirm that the surface sterilization was successful, 100 μL of the final rinsing water was cultivated on Luria Bertani (LB) medium plates, and the plates were examined for bacterial growth after incubation at 30°C for 48 h. For each replicate, three biological samples—biological bulk soil, rhizosphere soil, and endosphere—were analyzed as described below.

### DNA Isolation and 16S rDNA Sequencing to Assess the Bacterial Community

From samples collected from each replicate, DNA was extracted using the GeneAllExgene^TM^ Soil DNA isolation kit (GeneAll, South Korea) following the manufacturer’s instructions. The surface-sterilized roots were crushed with a sterilized mortar and pestle before DNA isolation (Garcias-Bonet et al., 2012). The isolated DNA was analyzed for purity using an Epoch^TM^ Spectrometer (BioTek, Vermont, United States), and DNA conditions were assessed by 1% agarose gel electrophoresis. To generate bacterial libraries, the first round of PCR was performed to amplify the V3-V4 region of the 16S rRNA gene using universal primers 341F (5′-CCTACGGGNGGCWGCAG-3′) and 805R (5′-GACTACHVGGGTATCTAATCC-3′)—which contains Nextera consensus and adaptor sequences at the forward (5′- TCGTCGGCAGCGTC-AGATGTGTATAAGAGACAG-target sequence-3′) and reverse (5′- GTCTCGTGGGCTCGG-AGATG TGTATAAGAGACAG-target sequence-3′) (Fadrosh et al., 2014). The PCR products were cleaned-up and amplified with primers containing Illumina dual indices and sequencing adapters using the forward index i5 (5′-AATGATACGGCGACCA CCGAGATCTACAC-55555555-TCGTCGGCAGCGTC-3′) and reverse i7 (5′-CAAGCAGAAGACGGCATACGAGAT-7777777-GTCTCGTGGGCTCGG-3′). The PCR conditions were as follows: Initial denaturation at 94°C for 3 min, followed by 25 cycles of denaturation at 94°C for 30 s, annealing at 55°C for 30 s, extension at 72°C for 30 s, and final extension at 72°C for 5 min. The PCR products were quantified using the Quant-iT PicoGreen dsDNA Assay Kit (Invitrogen, United States), and the quality was checked using an Agilent Bioanalyzer 2100 system. Purified amplicon libraries were pooled and sequenced at ChunLab Inc. (South Korea) with an Illumina MiSeq system using the MiSeq Reagent Kit v2 (Illumina Inc., United States).

### Sequencing Data Processing and Analysis

The sequenced data were analyzed using EzBioCloud^1^, an in-house pipeline developed by ChunLab Inc. (Seoul, South Korea) (Yoon et al., 2017). The pipeline provides quality control, merging of forward and reverse reads of paired-end reads, sequence processing and taxonomic classification, and diversity analysis of operational taxonomic units (OTUs). Low-quality raw sequencing reads were filtered out (average quality value < 25) using Trimmomatic v0.32. The paired-end sequences were merged using PandaSeq (Bolger et al., 2014), and primers were trimmed using the EzBioCloud program at a similarity cut-off of 0.8. The sequence errors were denoised using the DUDE-Seq software (Lee et al., 2017). The analysis pipeline uses the EzBioCloud 16S rRNA database, which removes chimeric sequences manually through UCHIME (Edgar et al., 2011). Taxonomic assignment was implemented by searching the EzBioCloud database using the USEARCH program (Edgar, 2013), and sequence similarity was calculated via pairwise alignment. Query sequences that matched the reference sequence at 3% sequence dissimilarity in EzBioCloud were considered identified at the species level. The sequences that did not match the database were clustered using Cluster Database at High Identity with Tolerance (CD-HIT) and UCLUST tools with 97% similarity (Li and Godzik, 2006). The species identified in the EzBioCloud database and OTUs obtained by CD-HIT and UCLUST were combined to form the final set of OTUs. The cutoff values for other reference sequences were as follows (× = similarity): Genus (97% > × ≥ 94.5%), family (94.5% > × ≥ 86.5%), order (86.5% > × ≥ 82%), class (82% > × ≥ 78.5%), and phylum (78.5% > × ≥ 75%) (Yarza et al., 2014).

Bacterial diversity was also analyzed and compared using CL community v3.43 (ChunLab, Seoul, South Korea) (Yi et al., 2014). The alpha diversity analysis, abundance-based coverage estimator (ACE), Chao1, Shannon, Simpson, and phylogenetic diversity indices as well as rarefaction curves were calculated. Differences in the alpha diversity—including the number of OTUs, richness, and diversity—were analyzed between the sites at which the samples were collected. Differences in taxonomic composition between the compartments (bulk soil, rhizosphere soil, and root) and sites were also compared from the phylum to species levels. The beta diversity, including principal coordinate analysis (PCoA) and UPGMA clustering, was analyzed based on the Bray-Curtis dissimilarity index (Bray and Curtis, 1957) at the species level. The relative differences of bacterial population between bulk soil, rhizosphere, and roots in respective habitat were determined using the least significant difference (LSD) test at *P* = 0.05. And the differences in respective compartments between wild and cultivated habitats at each taxonomic level were determined using the *t*-test at *P* = 0.05.

### Culture-Dependent Bacterial Estimation and Identification of Strains

General microbial populations were determined by dilution plating on nutrient-poor agar media. For enumeration of culturable microbes in bulk and rhizosphere soils, 5 g of soil from each compartment was weighed and added to 50 mL sterile distilled water (DW), vigorously stirred for 5 min, serially 10-fold diluted, and plated onto 1/10 Luria-Bertani (LB) agar media with 50 μg/mL cycloheximide for total bacterial counts. For isolation of endophytic bacteria, the roots were surface-disinfected as described above, ground in a sterilized mortar and pestle, and then suspended in sterile DW (10 mL/g tissue). The suspension was serially 10-fold diluted and plated onto 1/10 LB agar media. The plates were incubated at 30°C for 3 days, and the bacterial colonies were enumerated. The bacterial colonies showing distinctive colors and shapes were randomly picked and transferred individually to 1.5 mL tubes containing LB medium with 15% (v/v) glycerol. The tubes were then incubated at 30°C for 24 h and stored at –80°C for further biological analysis. To identify the strain, the 16S rDNA of the strain was sequenced using primers 27F and 1492R and aligned with the NCBI 16S rDNA sequences.

### Soil Physicochemical Analyses

The collected bulk soil samples were air-dried at room temperature in the shade and passed through a 2-mm sieve. The soil samples were mixed with DW at a ratio of 1:5 (w/v) for 30 min at 200 rpm, and the pH and electrical conductivity (EC) of the soils were determined using a pH meter (HI 9124, Hanna Instruments, United States) and EC meter (HI9033, Hanna), respectively. To determine the exchangeable cation content, 5 g of soil was mixed with 50 mL ammonium acetate (1 N, pH 7.0) solution for 30 min at 200 rpm and filtered to remove soil particles. The exchangeable Ca^2+^, K^+^, Na^+^, and Mg^2+^ contents were analyzed using inductively coupled plasma optical emission spectrometry (ICP-OES; 5800 ICP-OES, Agilent, United States) (Hwang and Son, 2006).

### Growth Promotion Assay of *Arabidopsis*

The bacterial strains, which had been isolated from the DPF plant and stored at–80°C, were revived in LB agar plates and incubated at 28°C for 48 h and analyzed for activity as plant growth promoting bacteria (PGPB). Bacterial cells were harvested from the plates in sterile DW (1 × 10^8^ CFU/mL) supplemented with 0.2% sterilized carboxymethyl cellulose (CMC). *Arabidopsis thaliana* ecotype Columbia-0 (Col-0) seeds were surface-sterilized with 70% (v/v) ethanol for 90 s and 1% (v/v) sodium hypochlorite for 5 min, followed by washing three times with sterile DW. Disinfected seeds were bacterized by soaking in each bacterial suspension. The suspensions were incubated at room temperature (approximately 25°C) in a rotary shaker at 150 rpm for 15 min to facilitate the attachment of bacterial cells to the seed coat. The seeds were placed in Petri dishes with sterilized filter papers to remove excess moisture. Seeds soaked in sterile DW amended with 0.2% CMC served as the control. After bacterization, *A. thaliana* seeds (5 seeds/plate) were sown onto Petri dishes (90 × 15 mm) containing half-strength Murashige and Skoog (1/2 MS) medium supplemented with 1.5% sucrose and 0.8% (w/v) agar. The plates were sealed with parafilm and placed at an angle of 70° in plant-growth chambers under light cycle (16-h light/8-h dark; 100 μmol m^–2^ s^–1^) conditions at 23 ± 1°C. After 10 days of cultivation, the length of the roots, number of lateral roots, root thickness, and fresh weight of the plants were measured (Bhattacharyya et al., 2015a). The experiment consisted of three replicates of five seeds each, and the entire experiment was repeated three times. The data were subjected to analysis of variance using SAS JMP software (SAS Institute, Cary, NC, United States). Significant differences in the treatment means of each sample were determined using the LSD test at *P* = 0.05. Data from each experiment were analyzed separately.

### Assays for Plant Growth Promoting and Stress Alleviating Activities

Siderophore production of each PGPB strain was determined using the modified chrome azurol S (CAS) agar method (Schwyn and Neilands, 1987). Briefly, the CAS solution mixed with 1 mM ferric chloride (FeCl_3_FeCl3) solution was added to hexadecyltrimethylammonium bromide (HDTMA) solution and autoclaved. The final mixture (100 mL) was added to 900 mL of autoclaved LB agar medium at pH 6.8. Bacterial cultures grown overnight in LB medium were spot-inoculated (20 μL) onto sterile paper discs laid on CAS plates, and the plates were incubated at 30°C for 4 days. The isolates exhibiting an orange halo were considered positive for siderophore production.

Phosphate solubilization ability was determined by inoculating each bacterial strain on Pikovskaya’s agar medium (Pikovskaya, 1948). After 3 days of incubation at 28°C, strains that produced a clear zone around the colonies were considered positive.

To determine indoleacetic acid (IAA) production, each bacterial strain was cultured in LB medium for 24 h at 30°C and then inoculated into the nutrient medium containing peptone (10 g), yeast extract (3 g), tryptone (0.5%), l-tryptophan (5 g), and DW (to 1.0 L) with cell concentration of 0.5 at OD_600_ nm (Apine and Jadhav, 2011). The cell-free supernatant was obtained by centrifugation (13,000 g, 10 min) after incubation at 30°C and 150 rpm for 48 h in the dark. The supernatant was mixed with Salkowski reagent (35% perchloric acid and 0.5 M ferric chloride) at a ratio of 3:2 and incubated for 30 min in the dark. IAA concentration was measured spectrophotometrically using a microplate reader (Infinite 200 Pro Tecan, Austria) at 530 nm. For cytokinin production, M9 medium (100 mL in 250 mL) supplemented with 0.2% casamino acids, 0.01% thiamine, and 2 μg of biotin per liter (Akiyoshi et al., 1987) was inoculated with 1 mL of culture and incubated at 28 ± 2°C and shaken at 160 rpm for 3 days. Cytokinin production was quantified spectrophotometrically at 665 nm (Patel and Saraf, 2017) using M9 medium as a control. The experiment was conducted in triplicate.

Extracellular protease production was determined by spot inoculation of each bacterial suspension on sterile filter paper discs placed on skim milk agar medium. Protease activity was identified by a clear zone at 3 days after incubation at 30°C. To determine the antimicrobial activities of the selected strains, which are useful characteristics for the suppression of biotic stresses, antibacterial activities against the bacterial pathogens *Xanthomonas oryzae* pv. *oryzae* and *Xanthomonas axonopodis* pv. *glycines* were tested using the dual inoculation technique (Kakembo and Lee, 2019). The antifungal activity of each PGPR strain against the mycelial growth of fungal pathogens, such as *Botrytis cinera* and *Fusarium oxysporum* was assayed using dual culture methods (Dutta et al., 2020). The experiment was replicated twice with three plates per replicate.

## Results

### Sample Preparation of DPF Plants From Wild Habitat in Karst Topography and Cultivated Areas

To study the features of microbial communities associated with the various compartments (bulk soil, rhizosphere soil, and root endosphere) of DPF plants in the wild habitat, we collected the endemic plants from the unique habitat of the karst topography in Jeongsun-gun, Kangwon-do province, South Korea (Supplementary Figure 1). The plants were primarily collected from limestone rocks (N37.35xxx and E128.63xxx) located at an altitude of 230–270 m (Supplementary Figure 1). Cracks between rocks at ridges or slopes are likely to be safe places for plants, having favorable light conditions and reduced competition for niche and nutrient resources (Oh et al., 2014). Furthermore, to compare the bacterial community structures between native wild habitats and cultivated areas, we sampled the DPF plants from the fields that are cultivated by the DPF Preservation Association^2^, which is also located in Kangwon-do province.

### Analysis of Illumina MiSeq Sequencing Data and Alpha Diversity

To investigate the characteristics of bacterial microbiota associated with DPF plants in wild and cultivated habitats, DNA was isolated from each rhizocompartment (bulk soil, rhizosphere soil, and endosphere), and 16S rDNA was sequenced. High-throughput sequencing generated a total of 1,335,577 (median 74,198) bacterial 16S rDNA reads, and the quality trimming, merging, and removal of chimeric reads produced a total of 647,498 reads with a median read length of 412.29. The rarefaction curve at 3% dissimilarity for bulk and rhizosphere soils showed an average of 5,826 and 5,788 OTUs, respectively, in the wild area and 4,657 and 4,310 OTUs in the cultivated area (Supplementary Figure 2 and Supplementary Tables 1, 2), which indicated that the sequencing depth was sufficient to cover detectable species both in bulk soil and rhizosphere samples. The number of OTUs from roots averaged 989 from the wild and 229 from cultivated areas, indicating that additional sequencing was needed to capture the complete diversity of the bacterial communities. However, considering many similar previous reports (Qiu et al., 2012), we concluded that the data were sufficient to compare the changes in bacterial diversity between bulk soil, rhizosphere, and root. Good’s coverage analysis revealed that more than 98% of the taxonomic richness was covered by sequencing in every sample of wild and cultivated areas, excluding root samples from cultivated areas where more than 95% of richness was covered (Supplementary Tables 1, 2).

**FIGURE 1:**
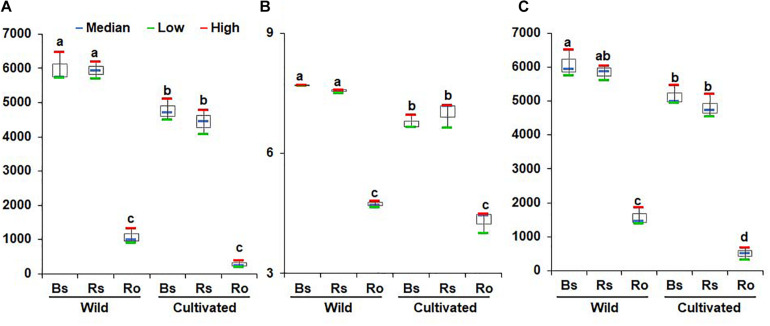
Comparison of richness and diversity of bacterial communities between rhizocompartments in wild habitats and cultivated areas. Richness was quantified by the Chao1 estimator **(A)**, species evenness was represented by Shannon’s index **(B)**, and biodiversity incorporating phylogenetic difference between species was measured through phylogenetic diversity **(C)**. Red and green bars represent high and low while horizontal blue bars within boxes represent median. The tops and bottoms of boxes represent the 75th and 25th quartiles, respectively. Diversities were compared between bulk soil (Bs), rhizosphere soil (Rs), and root (Ro) in wild habitats and cultivated areas. Different letter(s) indicate significant difference at P = 0.05.

Non-parametric analysis of diversity indices such as ACE, Chao1, and Jackknife indicated high bacterial diversity in wild habitats compared to cultivated areas (Figure 1A and Supplementary Tables 1, 2). The Shannon and phylogenetic diversity indices also indicated an increased abundance and richness of bacterial communities in the bulk soil, rhizosphere soil, and endosphere of wild habitat compared to cultivated areas (Figures 1B,C), indicating more ecological diversity in wild habitats. Among the different compartments in each area, the alpha diversity indices showed higher bacterial diversity in the bulk and rhizosphere soils than in the roots (Figure 1). Taken together, the bacterial community diversity was higher in the wild habitat than in the cultivated areas, indicating that intensive cultural management reduces microbial richness. In addition, bacterial diversity in the rhizosphere was higher than that in the endosphere, which is consistent with the findings of previous studies (Edwards et al., 2015; Pérez-Jaramillo et al., 2018).

### Difference in Bacterial Community Structure Based on Beta Diversity Analysis

**FIGURE 2:**
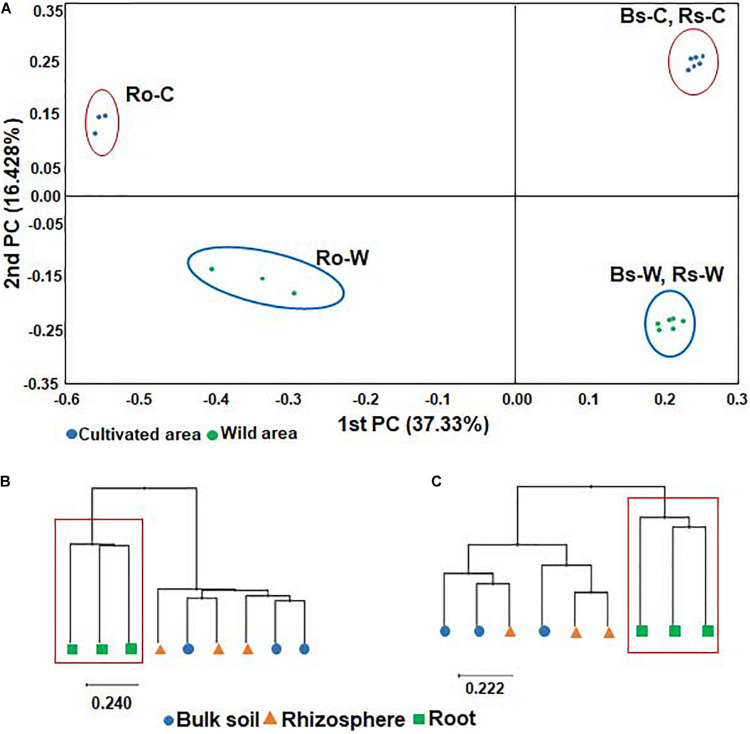
Principal coordinates analysis (PCoA) of bacterial microbiota **(A)** and UPGMA clustering based on Bray-Curtis dissimilarity in **(B)** wild and **(C)** cultivated areas using relative abundance of all OTUs. The red box shows the cluster formed exclusively in roots of both wild and cultivated habitats.

To compare the bacterial communities in soils and roots of DPF plants grown in wild habitats and cultivated areas, PCoA of all samples was performed based on the Bray-Curtis dissimilarity index. The beta diversity indices through PCoA revealed that the bacterial community of the wild habitat is significantly different from the corresponding compartments in cultivated areas. In both wild and cultivated areas, bacterial taxonomic structures and compositions of bulk and rhizosphere soils were similar, whereas the bacterial root endophyte microbiota was significantly different from that of the surrounding soils (Figure 2). The UPGMA clustering also showed that while the species-level bacterial community structures of the bulk and rhizosphere soils in each habitat were clustered together (i.e., there was a high representation of similar or related species in the two soil rhizocompartments), the bacterial community from the roots formed a distinctive cluster with that of the soils (Figure 2). The bacterial communities in the roots showed low species richness and evenness. Overall, the results indicate that the uniqueness of the bacterial composition of root endophytes might be selected by adaptation to the roots of DPF plants.

### Taxonomic Structure of Bacteria at the Phylum Level

**FIGURE 3:**
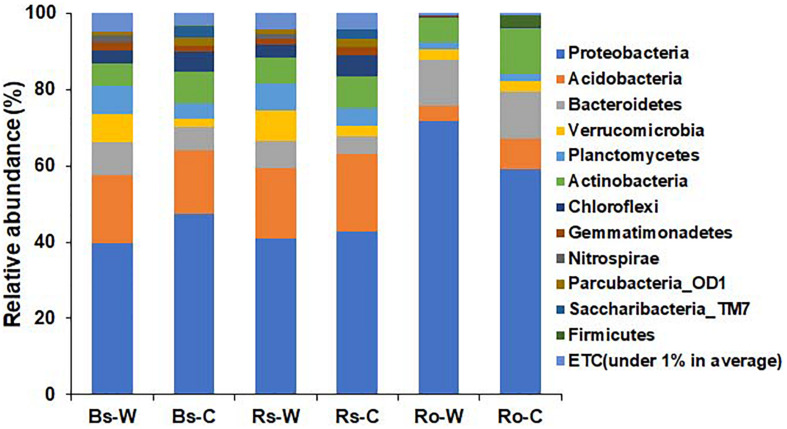
Comparison of bacterial composition and relative abundance at the phylum level in bulk soil (Bs), rhizosphere soil (Rs), and root (Ro) of long-sepal Donggang pasque-flower plant collected from wild (-W) and cultivated (-C) areas. A phylum with relative abundance less than 1% was referred to as “ETC”.

The bacterial profiles between the habitats and compartments of DPF plants were compared in terms of taxonomic levels. At the phylum level, the relative abundance of bacteria from wild and cultivated areas showed similar patterns in both bulk and rhizosphere soil samples (Figure 3). In the wild habitat, Proteobacteria were the most abundant in bulk and rhizosphere soils (39.8 and 41.0%, respectively), followed by Acidobacteria (17.8 and 18.4%, respectively). The abundance of Proteobacteria and Acidobacteria in the bulk and rhizosphere soils of cultivated areas was 47.3 and 42.9% and 16.9 and 20.2%, respectively, which is similar to the abundance detected in wild habitats. In the roots of DPF plants, the relative abundance of Proteobacteria in wild habitat (71.8%) was significantly higher than that (59.0%) of cultivated area. The abundance of Proteobacteria in roots of both areas was higher than that in the soils, followed by Bacteroidetes (12.10 and 12.14% in wild and cultivated area, respectively). Verrucomicrobia was highly abundant in wild rhizosphere, while Chloroflexi and Saccharibacteria_TM7 were significantly higher in rhizosphere of cultivated plants. In case of bulk soil, phyla Bacteroidetes, Verrucomicrobia and Planctomycetes were significantly higher in wild area while Saccharibacteria_TM7 was higher in cultivated habitat. Overall, Proteobacteria was the most abundant phylum in all rhizocompartments (Pérez-Jaramillo et al., 2018). Acidobacteria, Verrucomicrobia, Planctomycetes, and Chloroflexi were relatively abundant in bulk and rhizosphere soils compared to the root endosphere.

### Features of Bacterial Profiles at the Genus and Species Levels

**FIGURE 4:**
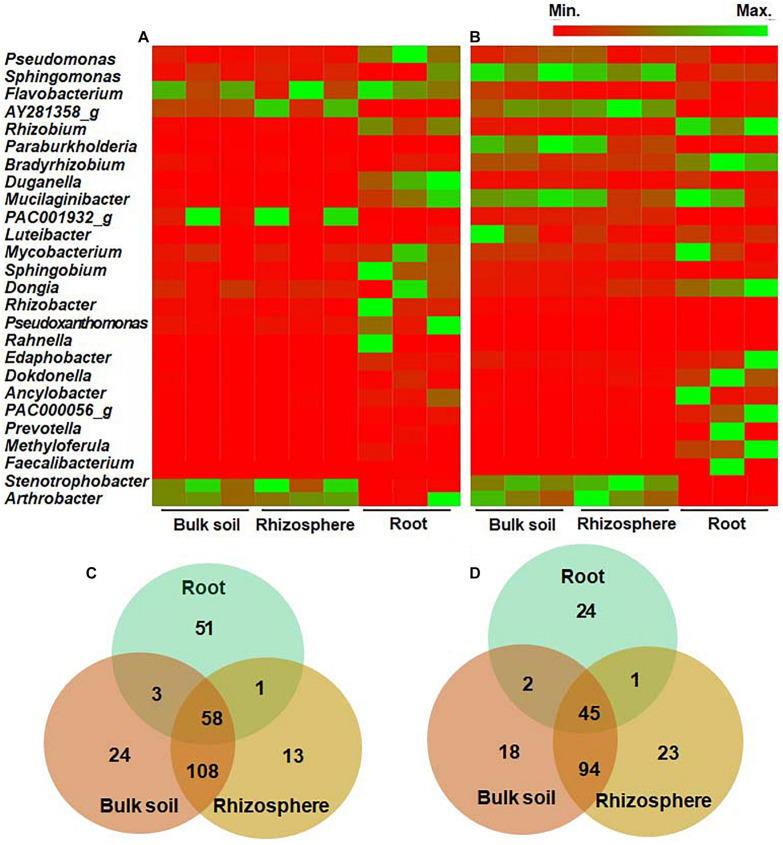
Comparison of relative abundance of bacteria at the genus level in bulk soil, rhizosphere, and root of long-sepal Donggang pasque-flower plants. The heat map analysis compared bacterial genera showing more than 1% relative abundance in surrounding soils and roots of wild **(A)** and cultivated **(B)** habitats. The Venn diagrams show unique or shared genera of bacterial microbiota with relative abundance above 0.1% in each rhizocompartment of wild **(C)** and cultivated **(D)** areas.

We further analyzed and compared the bacterial community structure at the genus and species levels. In the wild habitat, *Stenotrophobacter* and *Arthrobacter* were the most abundant genera in the bulk and rhizosphere soils of wild DPF plants (Figure 4A and Supplementary Figure 3). Most of the genera in bulk and rhizosphere soils (> 71 and > 69%, respectively) were below 1% of the average relative abundance, which indicates the presence of diverse bacteria with low abundance in the soils. Among the rhizosphere bacteria, 13 bacterial genera (∼8%) were distinguished from bulk soil, while 24 genera (∼13%)—such as *Cellvibrio, Erythrobacteraceae_uc, Flavisolibacter, Leifsonia, Pedobacter, Polaromonas, Rudrobacter*, and *Sphingopyxis*—were detected only in bulk soil (Figure 4). *Pseudomonas, Rhizobium, Pseudoxanthomonas*, and *Duganella* were the most abundant genera in the roots of wild plants. Among the genera with relative abundance > 0.1%, 51 genera (∼45%) of endophytes were distinctively distinguished from either bulk or rhizosphere soils, while 58 (∼51%) of them were shared with both bulk and rhizosphere soils.

In cultivated habitats, *Sphingomonas, Paraburkholderia*, and *Pseudomonas* were the most abundant genera in bulk and rhizosphere soils, while *Bradyrhizobium, Rhizobium*, and *Dongia* were the most abundant endophytes in the cultivated DPF plants (Figure 4B and Supplementary Figure 3). The genera showing less than 1% relative abundance in the bulk and rhizosphere was around 54 and 57%, respectively. Among the bacterial genera with more than 0.1% relative abundance, 18 (∼11%) and 23 (∼14%) genera in bulk and rhizosphere soils, respectively, were unique to each compartment (Figure 4), and 24 (33%) genera of endophytes in the roots of cultivated plants were also distinguished from soil compartments.

Bulk soils of wild and cultivated plants had 113 genera in common (> 0.1% abundance), such as *Bradyrhizobium, Mesorhizobium, Pseudomonas, Rhizobium, Sphingomonas, Sphingobium, Stenotrophobacter, Varribacter*, and *Variovorax* comprising 58.6 and 70.6%, respectively, of total identified bacteria in each habitat. A total of 128 genera (> 0.1% abundance), such as *Acidibacter, Bradyrhizobium, Caulobacter, Flvaobacterium, Nitrospira, Pseudomonas*, and *Rhizobium* were common between the rhizosphere of wild and cultivated areas, amounting to 71.1 and 78.1%, respectively, of the bacterial populations in each habitat. Between the roots of wild and cultivated plants, a total of 27 genera (> 0.1% abundance) were in common which amounted to 23.9 and 37.5%, respectively, of the endophytic bacterial community. Among the 58 and 45 genera identified in all rhizocompartments of wild and cultivated habitats, respectively, 24 genera such as *Bradyrhizobium, Caulobacter, Mesorhizobium, Pseudomonas, Rhizobium, Sphingobium, Sphingobium*, and *Variovorax* were shared by both habitats.

In summary, the relative abundance of bacteria at the genus level was similar between the bulk and rhizosphere soils of both wild and cultivated areas (Figure 4 and Supplementary Figure 3). However, the highly abundant genera in the roots were distinctively distinguished from those in the bulk and rhizosphere soils. The genera *Ancylobacter, Filimonas, Ideonalla, Kaista, Methyloferula, Prevotella*, and *Taibaiella* were the core endophytic bacteria in plants from both areas. The results indicated that the bacterial endophytes might be distinctively favored by the roots, and some of them were recruited from the surrounding environments.

**FIGURE 5:**
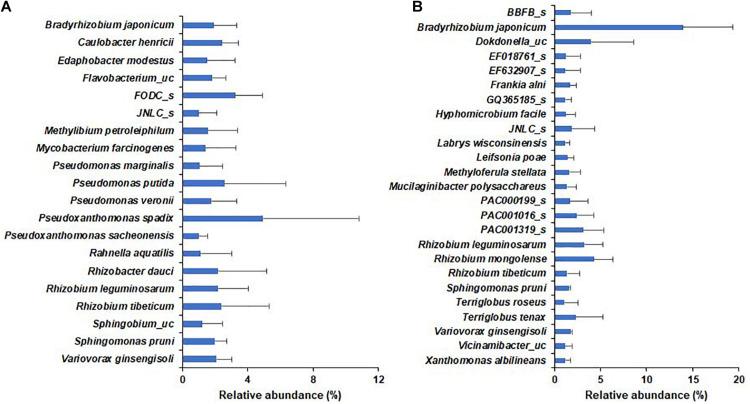
Relative abundance of bacterial species within the roots of long-sepal Donggang pasque-flower plants. The bacterial species with relative abundance > 1% in roots of Donggang pasque-flower plants were compared between wild **(A)** and cultivated **(B)** habitats.

In the wild habitat, all species identified in the bulk soil or rhizosphere compartments were below 1% average relative abundance, whereas 20 species, including *Pseudomonas putida* and nitrogen-fixing rhizobia, were above 1% average relative abundance in the roots of wild plants (Figure 5). In cultivated areas, 8 and 5 species in bulk and rhizosphere soil, respectively, were above 1% relative abundance (Supplementary Figure 4). The roots of the cultivated plants were abundantly enriched with 25 species, including nitrogen-fixing bacteria, such as *Bradyrhizobium japonicum* (13.98%), *Rhizobium*, and *Paraburkholderia* species. Among the species with more than 1% relative abundance, *Bradyrhizobium japonicum, Rhizobium leguminosarum, Rhizobium tibeticum, Sphingomonas pruni*, and *Variovorax ginsengisoli* were shared as root endophytes in both wild and cultivated plants (Figure 5) and there no significant difference in relative abundance of respective species among wild and cultivated habitat. Among bacterial genera with more than 0.1% relative abundance, the species of genera *Pseudomonas, Rhizobium, Novosphingobium, Pedobacter, Mesorhizobium, Ancylobacter, Flavobacterium, Sphingobium*, and *Sphingomonas* were more diverse in wild plants than in cultivated plants. Furthermore, the endophytes *Bradyrhizobium japonicum, Caulobacter henricii, Dokdonella ginsengisoli, Edaphobacter modestus, Filimonas lacunae, Herbiconiux ginseng, Hyphomicrobium facile, Ideonella azotifigens, Labrys methylaminiphilus, Labrys wisconsinensis, Leifsonia poae, Luteibacter rhizovicinus, Methyloferula stellata, Pseudomonas corrugata*, several *Rhizobium* spp. and *Variovorax* sp. were shared by both wild and cultivated plants; thus, these can be considered core endophytes of DPF plants.

Collectively, the results of this study revealed that bacterial diversity in the bulk and rhizosphere soils of wild habitats was higher than that of the corresponding rhizocompartments of cultivated habitat, indicating that the management of soils for the cultivation of plants reduces the diversity of bacteria. Our results also highlight that particular bacteria were distinctively selected and maintained as core endophytes in the roots of DPF plants irrespective of habitat, which strengthens the hypothesis that host factors are more important than environmental factors for the selection and maintenance of endophytes.

### Soil Properties

Cracks between rocks in the wild habitat of DSP plants hold fine soil particles for the plants to root into, although the soil structure is poor. The soils in the wild habitat were calcareous (> pH 7.5) with a high content of ionic calcium, which is characteristic of limestone karst regions (Supplementary Table 3). Soils of the cultivated areas were slightly acidic (approximately pH 6.7), and the magnesium and sodium content were higher than that of the wild areas. Li et al. (2018, 2019) suggested that the calcium content in karst regions is a major factor for endophytic bacterial community composition, which in turn helps the plants to adapt to the environment. Soil properties, such as pH, carbon-to-nitrogen ratio, available phosphorous, and potassium also determine the root microbial community directly or via plant growth (Lauber et al., 2009; Bulgarelli et al., 2012). Collectively, our results indicate that alkaline soils with high calcium content might influence the bacterial microbiota composition in the rhizosphere soil and roots of DPF plants, and the intrinsic bacterial strains isolated from the wild habitat might be developed for the growth of plants in distinctive habitats.

### Presence of Culturable Total Bacteria

Cultural management and soil properties induce changes in the nature and quantity of bacterial microbiota. In addition to profiling the bacterial community by 16S rDNA sequencing, we compared culturable bacterial numbers between the rhizocompartments of DPF plants collected from wild and cultivated habitats. In general, we could not observe any significant difference between wild and cultivated plants in terms of total culturable bacterial population in the bulk and rhizosphere soils (Supplementary Figure 5). In the case of endophytes, the roots of wild plants harbored higher bacterial populations than the roots of cultivated plants. The total culturable endophytes in the cultivated plants was lower than that in the bulk and rhizosphere soils. Our results indicate that there are no significant differences in the quantity of culturable bacteria between the surrounding soils of the wild and cultivated plants, and there is a comparatively low population of culturable bacteria in the root compared to the soil compartments.

### Plant Growth Promoting Activities of the Isolated Bacteria

**FIGURE 6:**
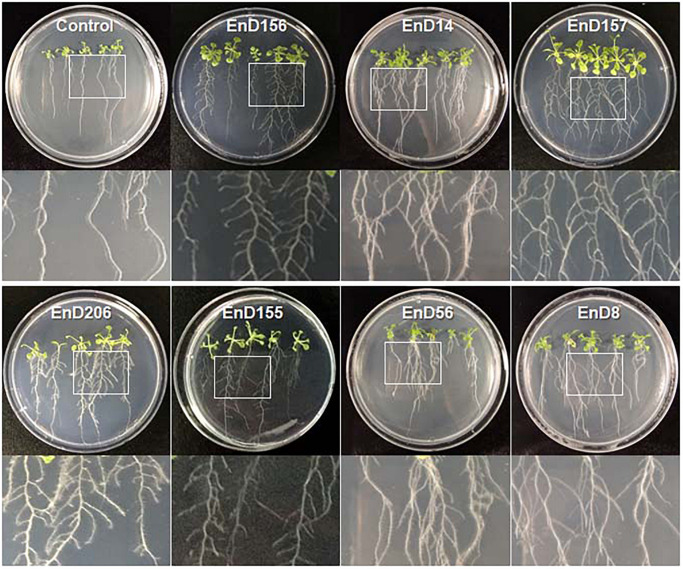
Growth promotion of *Arabidopsis* by treatment of bacterial cells isolated from rhizosphere and endophytic bacteria in long-sepal Donggang pasque-flower plants. *A. thaliana* ecotype Col-0 seeds were surface sterilized and bacterized by soaking in each bacterial suspension. The bacterized seeds were sown onto 1/2 MS medium and cultured in plant-growth chambers. The photographs shown were acquired 10 days after incubation. Refer to Table 1 for the scientific names of each strain.

Many rhizosphere- and root-dwelling bacteria have been reported to promote plant growth and suppress the activity of pathogens (Santoyo et al., 2016; Afzal et al., 2019). In this study, the bacterial strains isolated from the roots of DPF plants and the surrounding soils were assayed for their growth-promoting activity by bacterization onto *Arabidopsis* seeds. *Chryseobacterium wanjuense* EnD157, *Arthrobacter globiformis* EnD156, *Luteibacter rhizovicinus* EnD206, and *Phyllobacterium brassicacearum* EnD155 increased the fresh weight by 42.5, 15.7, 14.8, and 15.0%, respectively, and the number of lateral roots was significantly increased compared to the untreated control (Table 1, Figure 6, and Supplementary Figure 6). Among the strains, *A. globiformis* EnD156 and *C. wanjuense* EnD157 produced IAA and cytokinin, respectively, which may contribute to the increase in fresh weight. However, evidence of other specific growth-promotion activities, such as phosphate solubilization and siderophore production, was not detected. *Bacillus subtilis* EnD14, *Pseudomonas fluorescens* EnD56, and *Variovorax boronicumulans* EnD8 promoted root thickness growth with a high density of root hairs and the number of lateral roots (Table 1 and Figure 6). The strains *V. boronicumulans* EnD8, *B. subtilis* EnD14, and *P. fluorescens* EnD56 showed phosphate solubilization activity. The strains *B. subtilis* EnD14 and *V. boronicumulans* EnD8 produced phytohormones, and *P. fluorescens* EnD56 produced siderophores, which influence *Arabidopsis* growth (Supplementary Figure 7). In addition to the plant growth-promoting activity, *B. subtilis* EnD14 and *P. fluorescens* EnD56 showed antibacterial and antifungal activities as well as protease activity, indicating that these strains are candidates for the control of biotic stresses. Our assay indicated that nitrogen-fixing bacteria contribute slightly to the growth of *Arabidopsis* seedlings. However, further studies are needed for confirmation. Our results also indicated that different bacterial activities, such as phosphate solubilization and phytohormone production, produce various morphological variations in *Arabidopsis*, which requires further studies. We believe that these strains are potential microbial agents that can be developed for application in karst ecosystems.

**TABLE 1:** Plant growth promoting and antagonistic activities of bacteria selected from rhizosphere and roots of long-sepal Donggang pasque-flower plants.

Organism	Strain name	Origin^a^	Growth promotion activity^b^					Antagonistic activity^c^		
			GP	IAA	CK	PS	SP	Abac	Afun	Pase
*Arthrobacter globiformis*	EnD156	Bs-C	++	+	–	–	–	–	–	–
*Bacillus subtilis*	EnD14	Rs-W	+	–	–	+	–	+	+	+
*Chryseobacterium wanjuense*	EnD157	St-W	+++	–	+	–	–	–	–	+
*Luteibacter rhizovicinus*	EnD206	Rs-C	++	–	–	–	–	–	–	–
*Phyllobacterium brassicacearum*	EnD155	Bs-C	++	–	–	–	–	–	–	–
*Pseudomonas fluorescens*	EnD56	Bs-W	+	+	–	+	+	+	+	+
*Variovorax boronicumulans*	EnD8	Rs-W	++	+	–	+	–	–	–	–

*^a^Origin: Bulk soil (Bs), rhizosphere soil (Rs), and stem (St) from wild (-W) and cultivated (-C) areas. ^b^Growth promoting activity: GP, Growth promotion in terms of fresh weight of plants with significant differences determined using the least significant difference (LSD) test at P = 0.05 and designated as +++ >20%, ++> 20–10%, and +> 10–5%. IAA, indole acetic acid production; CK, cytokinin production; PS, Phosphate solubilization; SP, Siderophore production. ^c^Antagonistic activity: Abac, antibacterial activity against Xanthomonas oryzae pv. oryzae and X. axonopodis pv. glycines; Afun, antifungal activity against Botrytis cinera and Fusarium oxysporum; Pase, protease production.*

## Discussion

The DPF plant, an endemic and rare plant species of South Korea, is in danger of extinction, with low abundance and declining populations. They have restricted habitats in rocky areas, such as the ridges or slopes of karst topography (Korea Biodiversity Information System (KBIS), 2013; Oh et al., 2014). To save DPF species, government and private bodies have been attempting to propagate and transplant cultivated plants.

Microbial communities in the rhizosphere and plant tissues play important roles in plant physiological and metabolic processes as well as growth and yield (Bhattacharyya et al., 2015b; Bhattacharyya and Lee, 2016; Zahn and Amend, 2017). Understanding the rhizosphere and endophytic microbial community structures of DPF plants and their functions can contribute to the restoration and cultivation of plants. In this study, we analyzed the rhizosphere and endophytic bacterial microbiota of DPF plants growing in karst ecosystems and compared the microbial communities of cultivated DPF plants.

There were greater abundance and richness of bacterial microbiota in the bulk and rhizosphere soils of wild habitat DPF plants than cultivated area plants. The reduction in cultivated soil microbiota abundance might be due to cultural practices. Continuous cultivation practices and similar land use issues can lead to alterations in abiotic soil factors and cause the formation of similar soil properties in different fields, which in turn might affect the soil microbiome to form similar microbial community structures (Jangid et al., 2011; Tian et al., 2017). The complexity of microbiome interactions was reduced in agricultural soil compared to wild habitats (Pérez-Jaramillo et al., 2019) indicating that domestication of plants affects microbial community. Certain agricultural practices, such as integrated management of soil, have significantly reduced bacterial richness compared to soils managed organically and biodynamically, although the composition is similar (Hendgen et al., 2018). This study suggests that the soil microbiota needs to be diversified in cultivated fields for proliferation of plants.

While soil type is also an important driver of the microbial community composition in the rhizosphere, plant roots recruit and select specific microorganisms from the reservoir of microbiota in bulk soil to shape the composition of the microbiome in their rhizosphere (Tkacz et al., 2020). In this study, the bacterial microbiota in bulk soil and the rhizosphere were clustered together (in terms of biodiversity) for each of the wild and cultivated areas of DPF plants, despite significant differences in soil properties and vegetation between the two areas. Many studies have reported that the rhizospheres of different plant species or genotypes growing in the same soil develop distinct microbial communities, while some plant species develop similar communities in different soils (Miethling et al., 2000; Micallef et al., 2009). Therefore, the contribution or influence of the host is crucial in building the composition of the rhizosphere microbiome. The results of this study suggest that the microbial structure in the rhizosphere of the DPF plant might be established by the host plant.

Previous studies have found that the compositions of endophyte communities are highly dependent on plant roots as opposed to the soil types, and the same plant species tend to maintain similar endophytic composition irrespective of soil type (Afzal et al., 2019; Tkacz et al., 2020). Our results showed that endophytic bacterial diversity was lower than that of rhizosphere, which is consistent with previous reports (Edwards et al., 2015; Pérez-Jaramillo et al., 2018). Many studies have reported that plants selectively recruit a core microbiome independent of soil type, environment, host genotype, agricultural management, and other factors. These core microorganisms constitute a conserved subset of microbes that likely play important roles for host plants as well as for the surrounding microbial communities (Lundberg et al., 2012). In our study, known beneficial bacterial genera *Ancylobacter, Filimonas, Ideonalla, Kaista, Methyloferula, Prevotella*, and *Taibaiella* were identified as relatively abundant bacteria in the DPF roots of both habitats. Furthermore, several bacterial species were shared by both wild and cultivated plants. Recently, Padda et al. (2019) reported that the association between endophytic nitrogen-fixing bacteria and lodgepole pine trees was responsible for the survival of pine trees in gravel-mining sites. The presence of many nitrogen-fixing bacterial species belonging to *Bradyrhizobium, Rhizobium*, and *Mesorhizobium* as endophytes in both wild and cultivated DPF plants might contribute to the growth of the plants in harsh ecosystems in the karst region. The identification and functional elucidation of core microbiota of this study will be beneficial to develop biofertilizer for DPF plants in karst environment.

Soil type, pH, carbon-to-nitrogen ratio, and available P and K content influence the microbial community directly or indirectly by altering plant physiology (Lauber et al., 2009; Bulgarelli et al., 2012; Li et al., 2018). The DPF plants are primarily found in the cracks or in between rocks, which are likely to be ideal places for plants owing to favorable light conditions, some soil particles to root into, and low competition for space and nutrients (Oh et al., 2014). The microhabitat soils of wild DPF plants were calcareous (> pH 7.5) but with low content of magnesium and sodium compared to the cultivated soils. A plant’s intake of calcium is related to the amount of exchangeable calcium in soil and high calcium content in soil can lead to reduced photosynthesis and transpiration of plants (Li et al., 2020). The properties of soil also affected the root morphology and root exudation (Neumann et al., 2014), which are crucial factors for surrounding bacterial community. The changes of plant physiology modulated the endophytes and soil biological activities (Jones et al., 2009; Li et al., 2018, 2019). Therefore, properties of the calcareous soil might restrict the growth of sensitive plants and can also influence the composition of distinctive bacterial microbiota that may contribute to the adaptation of DPF plants to the karst ecological environment. A previous report indicated that the available mineral nutrients, especially magnesium was crucial for plant development via nitrogen-fixing bacteria, such as *Bradyrhizobium* and *Rhizobium* (Niu et al., 2015; Egamberdieva et al., 2018). The availability of mineral nutrients for DPF plant growth and the endophyte communities, including nitrogen-fixing bacteria, requires further study.

Systematic research into the associated microbiomes in endangered plants will contribute to the conservation of the plants in the wild as well as to the development of better cultivation (Fernández-Gómez et al., 2019; Dastogeer et al., 2020). In this study, we isolated bacterial strains from the soils and roots of DPF plants collected from both natural limestone habitats and cultivated areas for restoration and exhibition. Some strains, such as *Arthrobacter globiformis, Bacillus subtilis, Chryseobacterium wanjuense, Luteibacter rhizovicinus, Phyllobacterium brassicacearum*, and *Pseudomonas fluorescens* promoted *Arabidopsis* growth. Most of the strains have been previously reported to be associated with plant growth-promoting activity (Egamberdieva et al., 2018; Afzal et al., 2019). For instance, in addition to the well-known plant growth-promoting species of *Pseudomonas* and *Bacillus*, *Variovorax boronicumulans* has been reported to colonize the root tissues of orchids along with mycorrhiza and contribute toward seedling development at early stages (Herrera et al., 2020). The abundance of plant-beneficial bacteria associated with DPF plants might contribute to the survival and establishment of host plants in the natural environment.

Plant growth-promoting bacterial strains benefit plants mainly through nitrogen fixation, phosphate solubilization, and phytohormone and siderophore production. The strains selected in this study promoted *Arabidopsis* growth through phytohormone production and phosphate solubilization. *A. globiformis* EnD156 and *C. wanjuense* EnD157 produced IAA and cytokinin, respectively, while the strains *V. boronicumulans* EnD8, *B. subtilis* EnD14, and *P. fluorescens* EnD56 solubilized phosphate. Intriguingly, the growth metrics of *Arabidopsis*, such as lateral root number, root length, and shoot growth, varied depending on the identified mechanisms for growth promotion, which needs further study to understand microbial and plant interactions. The increase in number of lateral roots can influence the plant growth by improving water and nutrient uptake (Azranesh et al., 2011) under drought stress, which is a characteristic environmental condition in the high-calcium rocky regions. The production of IAA by rhizobacteria increased the number of lateral roots (Dimkpa et al., 2009). Our strains inducing later root formation by producing IAA can benefit in water stressed conditions of karst region. The nitrogen-fixing ability slightly contributed to the growth promotion of *Arabidopsis* but needs further study for confirmation. In addition, *B. subtilis* EnD14 and *P. fluorescens* EnD56 showed antibacterial, antifungal activity, and protease activities, which are potent characteristics of biocontrol agents. The strains isolated in this study could be used for the development of bacterial agents for the stable growth of plants, including DPF plants, in karst ecosystems. The results of this study will contribute to the design and implementation of effective microbiomes for the conservation of endangered plants.

In this study, we identified the bacterial microbiota associated with DPF plants in the wild habitat of karst ecosystems and compared the bacterial community structure with that of cultivated DPF plants. We also examined the core bacterial species of DPF plants by comparing the bacterial microbiota from both habitats. To the best of our knowledge, this study is the first to report the rhizosphere and endophyte bacterial community structures associated with DPF plants in the karst ecological environment. The bacterial strains associated with plant growth-promoting activity in this study could be used for the sustainable growth and restoration of rare and endangered plants in karst ecosystems. However, the mechanism through which the DPF plant, a rare and endangered species in karst topography, adopts microbes in the specific niche remains to be revealed. We expect further comparison of microbiota between wild and cultivated DPF plants to elucidate the role of domestication in depleting plant-associated microbiota. The contribution of each bacterial strain or bacterial consortium in the adaptation of the DPF plants to grow in soils with high calcium content or stress alleviation in the harsh ecosystem will be studied in the near future. We will also study further to develop bioformulations for the application of the potential strains.

## Data Availability Statement

The datasets presented in this study can be found in online repositories. The names of the repository/repositories and accession numbers can be found below: (NCBI PRJNA704228, PRJNA706074, PRJNA715571, PRJNA716746, PRJNA716744, and PRJNA717056).

## Author Contributions

SD performed the experiments, analyzed the data, prepared figures and tables, and drafted the manuscript. CSN contributed sampling and reviewed drafts of the manuscript. YHL contributed to the design, analysis and interpretation of data and revised critically the manuscript. All authors approved the submitted version.

## Conflict of Interest

The authors declare that the research was conducted in the absence of any commercial or financial relationships that could be construed as a potential conflict of interest.

## Footnotes

1 https://www.ezbiocloud.net/

2 http://www.idonggang.com/
