# Supplementary material for: Features of Bacterial Microbiota in the Wild Habitat of Pulsatilla tongkangensis, the Endangered “Long-Sepal Donggang Pasque-Flower Plant,” Endemic to Karst Topography of Korea
Source: Front Microbiol. 2021 Jul 8;12:656105. doi: 10.3389/fmicb.2021.656105 (PMC8297415; doi:10.3389/fmicb.2021.656105)
Supplement: Supplementary file 1 [file Data_Sheet_1.docx]

# Supplementary Material

# Supplementary Figures and Tables

## Supplementary Figure

*
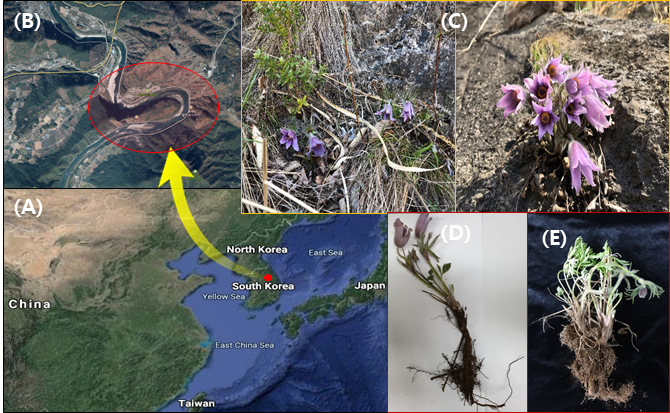
*

**Figure 1.** Sampling sites and long-sepal Donggang pasque-flower plant. The location of the endemic plant distribution (red dot) in karst topography of Kangwon-do province, South Korea (A and B). The plants were mostly collected from cracks between rocks (C) of slopes at the Donggang River located at an altitude of 200-300 m (red circle in B). The plants were collected from native (D) and cultivated (E) areas for microbe profiling.


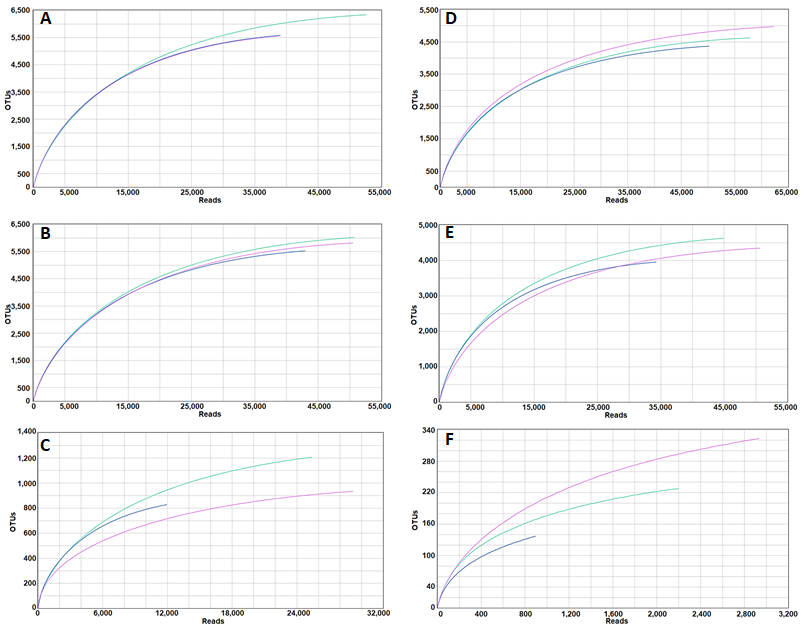


**Figure 2.** Rarefaction curves for bacterial operational taxonomic units (OTUs) of long-sepal Donggang pasque-flower plant. Rarefaction curves of bulk soil (A, D), rhizosphere (B, E), and endophytes (C, F) in wild habitats and cultivated areas, respectively, with the cut-off value at 97% similarity. The vertical axis indicates the number of OTUs expected after sampling the number of sequences denoted in the horizontal axis.


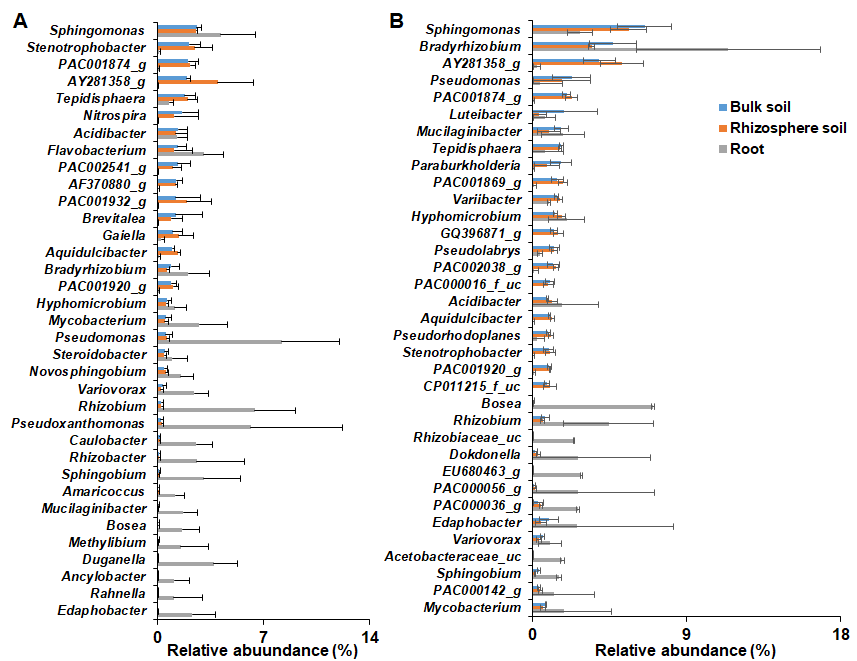


**Figure 3.** Relative abundance (%) of bacterial genera in bulk soil, rhizosphere, and roots of Donggang pasque-flower plants. The bacterial genera with relative abundance above 1% in each rhizocompartment of (A) wild and (B) cultivated habitats were compared. Bars illustrate the mean relative abundance (>1%) per compartment of each dominant phylum ± standard deviation.


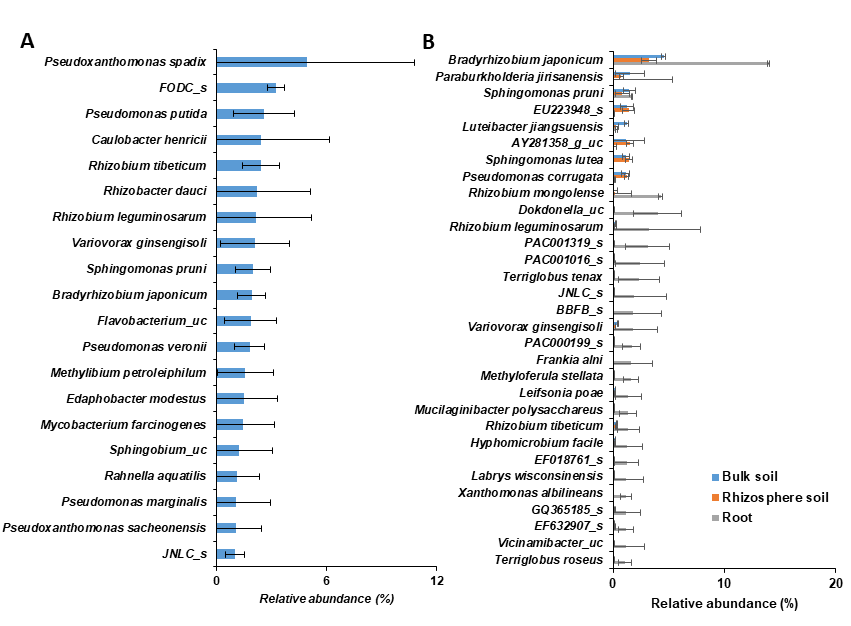


**Figure 4.** Relative abundance of bacterial species in bulk soil, rhizosphere, and root of long-sepal Donggang pasque-flower plants. The bacterial species with relative abundance >1% were compared between bulk soil, rhizosphere, and root of wild (A) and cultivated (B) habitats. Note: since none of the identified species exceeded 1% in bulk soil and rhizosphere of the wild area, (A) shows species detected in roots.


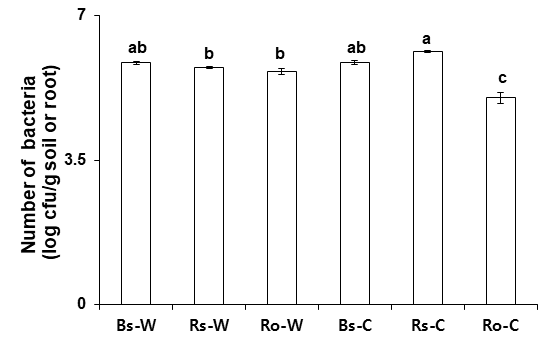


**Figure 5.** Total number of culturable bacteria in rhizosphere and root of long-sepal Donggang pasque-flower plant. Bacterial colonies were assessed from bulk soil (Bs), rhizosphere soil (Rs), and root (Ro) of long-sepal Donggang pasque-flower plants collected from wild (-W) and cultivated (-C) areas. Data presented as the mean ± SD, and bars with same letter(s) do not differ significantly at *P*=0.05.


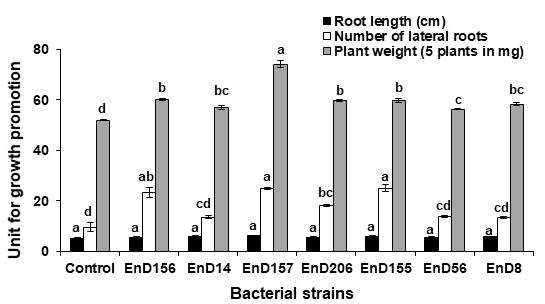


**Figure 6.** Effect of selected strains on growth of *Arabidopsis thaliana*. The seeds of *A. thaliana* Col-0 were bacterized with each strain and placed on half-strength MS medium for 10 days. Data represent the average ± SD of root length, number of lateral roots, and fresh weight of plants. Bars with same letter(s) do not differ significantly at *P*=0.05. The experiment was performed three times, each with three replicates, and each replicate consisted of five plants each. Note: Refer to Table 1 for scientific names of each strain.


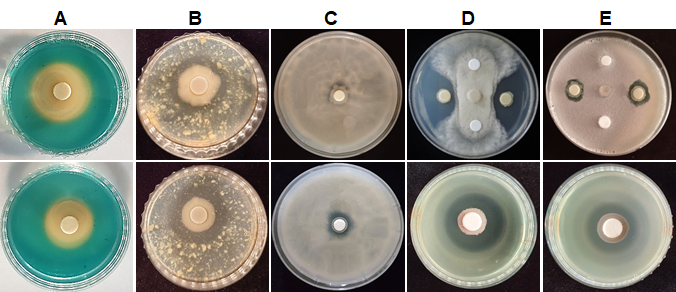


**Figure 7**. Growth-stimulating and antimicrobial activities of bacterial strains isolated from the rhizosphere and root of long-sepal Donggang pasque-flower plants. Siderophore production (A) was assessed from *Pseudomonas fluorescens* strains EnD56 and EnD210 (from upper to low panel) based on a change in the color of CAS medium from blue to orange. Protease activity (B) of the strains *Bacillus subtilis* EnD14 and *P. fluorescens* EnD56 was determined using skim milk agar medium based on the presence of a clear zone around colonies. Phosphate solubilization (C) of the strains *B. subtilis* EnD14 and *P. fluorescens* EnD56 was determined using Pikovskaya’s agar medium based on the formation of a clear zone around the colonies. Antagonistic activity of *B. subtilis* EnD-14 (D) against the fungal pathogen *Botrytis cinerea* and bacterial pathogen *Xanthomonas oryzae* pv. *oryzae.* Antifungal and antibacterial activity of *P. fluorescens* EnD56 (E) against *Pyricularia oryzae* and *Xanthomonas axonopodis* pv. *glycines,* respectively.

## Supplementary Tables

Table 1. Estimated OTU richness and diversity indices in the bulk soil, rhizosphere soil, and roots from plants in wild areas

| Sample | | Valid reads | OTUs | Ace | Chao1 | Jackknife | Shannon | Simpson | Goods Library Coverage (%) |
| --- | --- | --- | --- | --- | --- | --- | --- | --- | --- |
| Bulk soil | 1 | 52,606 | 6,340 | 6663.7 | 6470.0 | 7,035 | 7.70 | 0.001 | 98.68 |
|  | 2 | 39,040 | 5,584 | 5969.5 | 5762.1 | 6,347 | 7.71 | 0.001 | 98.05 |
|  | 3 | 38,833 | 5,553 | 5927.8 | 5728.4 | 6,308 | 7.71 | 0.001 | 98.06 |
| Rhizosphere soil | 1 | 50,683 | 6,019 | 6399.6 | 6190.1 | 6,786 | 7.60 | 0.002 | 98.49 |
|  | 2 | 42,987 | 5,528 | 5931.2 | 5713.9 | 6,301 | 7.58 | 0.002 | 98.20 |
|  | 3 | 50,513 | 5,818 | 6124.2 | 5943.8 | 6,469 | 7.52 | 0.002 | 98.71 |
| Root | 1 | 25,393 | 1,206 | 1393.3 | 1325.5 | 1,476 | 4.73 | 0.034 | 98.94 |
|  | 2 | 11,950 | 828 | 963.9 | 912.4 | 1,023 | 4.66 | 0.044 | 98.37 |
|  | 3 | 29,209 | 934 | 1055.3 | 999.9 | 1,109 | 4.82 | 0.022 | 99.40 |

Table 2. Estimated OTU richness and diversity indices in the bulk soil, rhizosphere soil, and roots from plants in cultivated areas

| Sample | | Valid reads | OTUs | Ace | Chao1 | Jackknife | Shannon | Simpson | Goods Library Coverage (%) |
| --- | --- | --- | --- | --- | --- | --- | --- | --- | --- |
| Bulk soil | 1 | 50,217 | 4,370 | 4,637.5 | 4,508.7 | 4,914 | 6.67 | 0.008 | 98.92 |
|  | 2 | 62,273 | 4,976 | 5,249.5 | 5,113.4 | 5,546 | 6.96 | 0.005 | 99.08 |
|  | 3 | 57,852 | 4,626 | 4,837.4 | 4,712.0 | 5,085 | 6.67 | 0.009 | 99.21 |
| Rhizosphere soil | 1 | 44,946 | 4,627 | 4,932.1 | 4,784.3 | 5,229 | 7.19 | 0.003 | 98.66 |
|  | 2 | 34,290 | 3,955 | 4,215.4 | 4,084.4 | 4,467 | 7.21 | 0.003 | 98.51 |
|  | 3 | 50,673 | 4,348 | 4,588.3 | 4,460.1 | 4,848 | 6.64 | 0.012 | 99.01 |
| Root | 1 | 897 | 137 | 201.3 | 213.6 | 353.1 | 4.03 | 0.032 | 94.09 |
|  | 2 | 2,934 | 323 | 408.0 | 408.4 | 443.0 | 4.50 | 0.028 | 96.59 |
|  | 3 | 2,202 | 228 | 282.0 | 266.5 | 288 | 4.46 | 0.023 | 97.28 |

Table 3. Physiochemical properties of soils

| Area  (Replication) | Properties | | | | | |
| --- | --- | --- | --- | --- | --- | --- |
|  | pH | Electrical conductivity  (dS/m) | Ca^2+^  (cmol/Kg) | K^+^  (cmol/Kg) | Mg^2 +^  (cmol/Kg) | Na^+^  (cmol/Kg) |
| Wild area | | | | | | |
| 1 | 7.67±0.02 | 0.60±0.01 | 15.47±0.01 | 0.45±0.01 | 1.25±0.01 | 2.04±0.06 |
| 2 | 7.57±0.03 | 0.66±0.00 | 13.77±0.37 | 0.52±0.00 | 1.39±0.03 | 1.52±0.05 |
| 3 | 7.70±0.04 | 0.60±0.01 | 14.01±0.07 | 0.42±0.01 | 1.18±0.00 | 1.88±0.04 |
| Cultivated area | | | | | | |
| 1 | 6.73±0.04 | 0.53±0.02 | 8.09±0.10 | 0.26±0.01 | 1.56±0.00 | 5.69±0.13 |
| 2 | 6.75±0.01 | 0.43±0.00 | 8.21±0.01 | 0.27±0.02 | 1.63±0.01 | 5.25±0.18 |
| 3 | 6.76±0.01 | 0.42±0.04 | 10.35±0.03 | 0.44±0.01 | 2.43±0.01 | 3.48±0.08 |
